# Wanted: Copies of January Number of the Homœopathic Physician

**Published:** 1881-09

**Authors:** 


### WANTED,

Copies of the January number of this Journal: thirty cents each will be paid for the same. Gentlemen wishing to dispose of this number, will please forward to 2109 Chestnut Street, Philadelphia. Write your name on outside of wrapper.

---

### CHANGE OF HEART.

We are glad to see that the *American Observer* has experienced a change of heart. In its July issue, instead of abusing pure homœopathy, as has been its habit, it reviews the *Revised New Testament*. Doubtless a *new* book to them!

